# The *Capsicum baccatum*-Specific Truncated NLR Protein CbCN Enhances the Innate Immunity against *Colletotrichum acutatum*

**DOI:** 10.3390/ijms22147672

**Published:** 2021-07-18

**Authors:** Seungmin Son, Soohong Kim, Kyong Sil Lee, Jun Oh, Inchan Choi, Jae Wahng Do, Jae Bok Yoon, Jungheon Han, Sang Ryeol Park

**Affiliations:** 1 National Institute of Agricultural Sciences, Rural Development Administration, Jeonju 54874, Korea; linewind@korea.kr (S.S.); island1984@naver.com (S.K.); golderic@naver.com (K.S.L.); osculation@korea.kr (J.O.); inchchoi@korea.kr (I.C.); jungheon1@hanmail.net (J.H.); 2 Pepper and Breeding Institute, K-Seed Valley, Gimje 54324, Korea; wahng0@hanmail.net (J.W.D.); jaebokyoon@hanmail.net (J.B.Y.)

**Keywords:** anthracnose resistance, *Capsicum baccatum*, *Colletotrichum acutatum*, *Nicotiana benthamiana*, nucleotide-binding and leucine-rich repeat

## Abstract

Chili pepper (*Capsicum* *annuum*) is an important fruit and spice used globally, but its yield is seriously threatened by anthracnose. *Capsicum baccatum* is particularly valuable as it carries advantageous disease resistance genes. However, most of the genes remain to be identified. In this study, we identified the *C. baccatum*-specific gene *CbCN*, which encodes a truncated nucleotide-binding and leucine-rich repeat protein in the anthracnose resistant chili pepper variety PBC80. The transcription of *CbCN* was greater in PBC80 than it was in the susceptible variety An-S after *Colletotrichum acutatum* inoculation. In order to investigate the biological function of CbCN, we generated transgenic tobacco lines constitutively expressing *CbCN*. Notably, *CbCN*-overexpressing transgenic plants exhibited enhanced resistance to *C. acutatum* compared to wild-type plants. Moreover, the expression of pathogenesis-related (*PR*) genes was remarkably increased in a *CbCN*-overexpressing tobacco plants. In order to confirm these results in chili pepper, we silenced the *CbCN* gene using the virus-induced gene silencing system. The anthracnose resistance and expressions of *PR1*, *PR2*, and *NPR1* were significantly reduced in *CbCN*-silenced chili peppers after *C. acutatum* inoculations. These results indicate that CbCN enhances the innate immunity against anthracnose caused by *C. acutatum* by regulating defense response genes.

## 1. Introduction

New breeding technologies comprising genomics approaches have been considered to be the primary method for screening for novel genes. Indeed, in consideration of the information in the genome sequence and the development of genome engineering technologies, genomics techniques such as genome-wide comparative analysis, quantitative trait locus (QTL), and map-based cloning constitute rapid and efficient methods for identifying valuable novel genes. Previous studies on the genome sequence of chili pepper (*Capsicum annuum*) revealed that the plant pathogen resistance (*R*) genes belonged to the nucleotide-binding and leucine-rich repeat (NLR) family are significantly expanded and diversified in the genome compared to closely related species [1]. In addition, in-depth genome-wide comparison showed that long terminal repeat retrotransposons dramatically elevated the numbers of NLR genes in chili pepper plants [2]. The NLR protein family regulates the immune response against invading pathogens in plants and animals [3,4,5]. Most *R* genes encode NLR receptors, which directly recognize pathogen effectors by the decoy domain [6]. This NLR-mediated effector triggered immunity commonly results in a hypersensitive response characterized by a burst of reactive oxygen species and programmed cell death associated with disease resistance in the infected region [7,8,9]. It also causes a secondary resistance response known as systemic acquired resistance (SAR), which is a mechanism of induced defense that confers long-lasting protection against a broad spectrum of pathogens [10].

The NLR family is one of the most variable gene families in plant genomes [11]. Plant NLRs are classified in two groups based on their N-terminal domains: toll/interleukin-1 receptor (TIR) domain containing NLRs (TNLs), coiled-coil (CC) domain containing NLRs (CNLs), and resistance to powdery mildew 8 (RPW8)-type CNLs (RNLs) [12]. NLRs also display a central nucleotide-binding site (NBS), which is also called NB-ARC, and a C-terminal leucine-rich repeat (LRR) domain [13]. In addition to these typical NLRs, atypical truncated NLRs lacking the LRR domain, namely TIR-NBS (TN) and CC-NBS (CN), are found in higher plant genomes [14,15]. Previous studies have demonstrated a biological function of TNs in *Arabidopsis*. For example, resistance to *Leptosphaeria maculans* 3 (RLM3), which encodes a TN protein, enhances the resistance to several necrotrophic fungi [16]. The truncated TN protein *Arabidopsis* chilling sensitive 1 (CHS1) plays a role in plant growth and cell death under chilling stress [17]. TN2 interacts with a subunit of the exocyst complex EXO70B1 and activates defense responses and cell death in the *exo70B1* mutant [18]. TN13 identified as an interaction partner of importin-α3/modifier of SNC1, 6 (MOS6) is required for basal resistance to the *Pseudomonas syringae* pv. *tomato* strain DC3000 [19]. The TIR-NB-LRR immune receptor suppressor of CHS1, 2, 3 (SOC3) interacts with CHS1 or TN2 and the SCF complex to regulate innate immunity [20,21]. Recently, the singleton TN, dangerous mix 10 (DM10), was reported to cause severe hybrid necrosis, which is typically induced by a conflict between divergent alleles of immunity genes coming from different parental accessions [22]. Although these data implicate their involvement in innate immunity, the roles of truncated NLRs, especially CN proteins, remain to be elucidated [23].

Chili pepper is a primary economic crop used as vegetable food, spice, and traditional medicine in the world [24,25,26] and products of it are severely threated by anthracnose which can cause product losses of up to 80% [27]. *Capsicum baccatum* contains a lot of valuable genes for pepper plant breeding [28,29]. In particular, the *C. baccatum* resistant variety PBC80 showed disease resistance to the three main *Colletotrichum* species causing pepper anthracnose: *C. acutatum*, *C. capsici*, and *C. gloeosporioides* [30]. The resistance genes originating from variety PBC80 have therefore been the focus of much attention toward their identification and characterization.

Previously, we generated the BC1F2 population by the interspecific crossing of *C. annuum* ‘SP26’ x *C. baccatum* ‘PBC80’. BC1F2 population showed enhanced anthracnose resistance to *Colletotrichum* *acutatum* and *Colletotrichum* *capsici* [31]. A composite interval mapping (CIM) analysis revealed main-effect QTLs for anthracnose resistance in a BC_1_F_2_ population and significant QTLs for resistance to *C. acutatum* located on chromosome 12 [31]. In this study, we cloned the candidate genes located on chromosome 12 from the *C. baccatum* variety PBC80 and identified *CbCN,* which is the *C. baccatum*-specific truncated *NLR* gene, by using a genome-wide comparative analysis. We took an integrated approach combining molecular and genetic tools to decipher *CbCN*’s biological function. Our findings revealed that *CbCN* is involved in innate immunity against anthracnose and regulates the expression of immune response genes.

## 2. Results

### 2.1. Identification of Truncated NLR Protein Specific of C. baccatum in the PBC80 Variety

**Figure 1:**
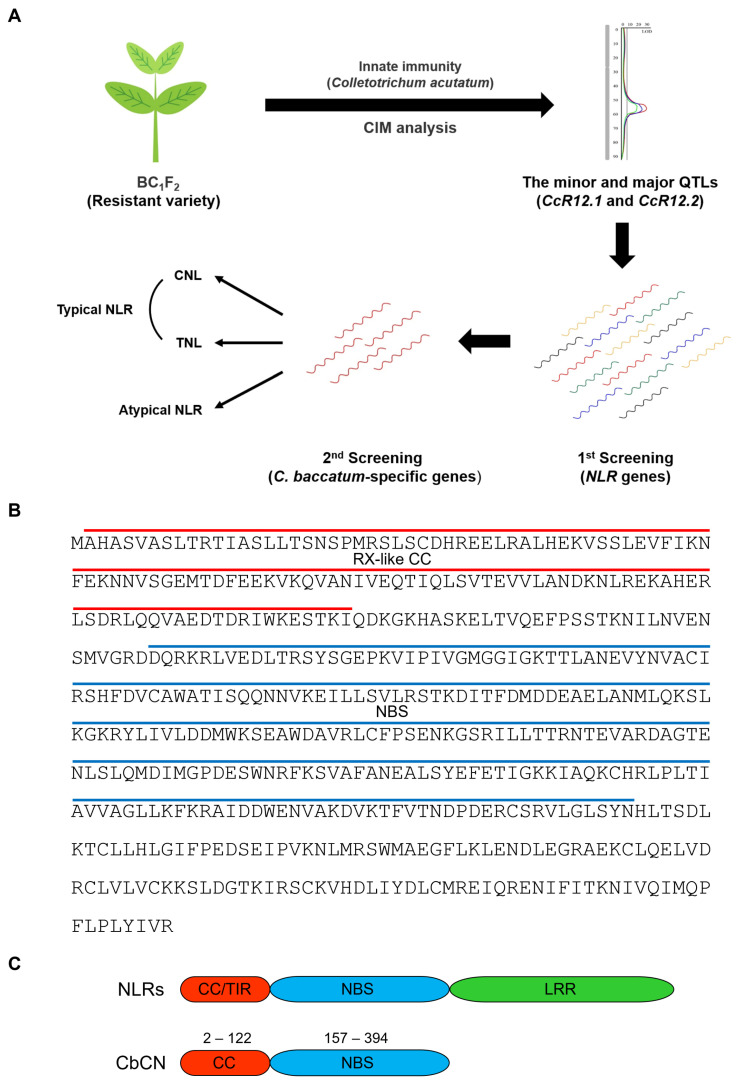
Consensus sequences of the atypical NLR protein CbCN. (**A**) Schematic representation of identification of the *CbCN* gene by genomic analysis. (**B**) Amino-acid sequences of CbCN cloned from PBC80. The 508 amino-acid sequences of the CbCN protein were analyzed and conserved domains were predicted using the InterPro program. The red line indicates the CC domain and the blue line indicates the NBS domain. (**C**) Schematic representation of a typical NLR and CbCN protein. The numbers correspond to the amino acids of the CbCN protein.

Our previous CIM analyses found the minor and major QTLs, *CcR12.1* and *CcR12.2*, located on chromosome 12 for resistance to *C. acutatum* [31]. Since *C. baccatum* contains a number of valuable genes for plant breeding, we performed a genomic analysis to find *C. baccatum*-specific *NLR* genes of the target region of two QTLs (Figure 1A). As a result, we identified the five *C. baccatum*-specific genes encoding NLR protein located on *CcR12.1* and *CcR12.2* (data not shown). In order to explore the function, we cloned the candidate genes from the resistant chili pepper variety PBC80. The cDNA sequence of one of them contained 1527 bp encoding a 508 aa protein lacking two asparagine residues (position 217 and 218) compared to the sequence of reference genome (PHT28388). The InterPro protein sequence analysis and classification software tool identified a RX-like CC domain spanning residues 2–122 and a NBS domain formed by residues 157–394 (Figure 1B). In contrast to typical NLR proteins, the identified protein presented only CC and NBS domains, while the LRR domain was absent (Figure 1C).

### 2.2. Upregulation of CbCN Transcription by C. acutatum

**Figure 2:**
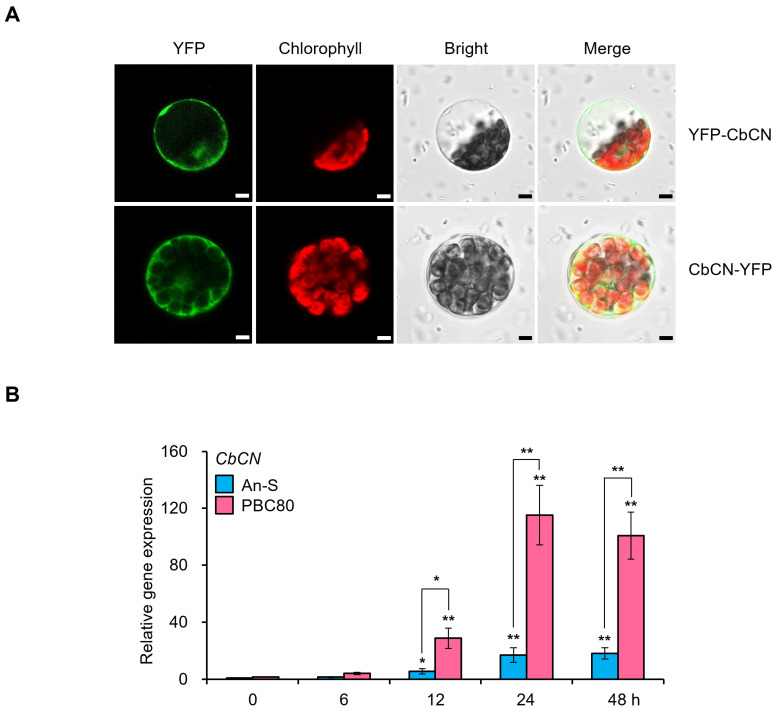
Analysis of CbCN subcellular localization and gene expression. (**A**) Subcellular localization of CbCN. N-terminal and C-terminal YFP-conjugated CbCN were transfected into chili pepper protoplasts and incubated for 12 h. Images were taken with a confocal microscope after the incubation. RFP marks the chlorophyll. Scale bar: 10 mm. (**B**) Expression analysis of *CbCN* after *C. acutatum* inoculation for the indicated times using RT-qPCR. Actin served as a control. Values represent means ± SDs. Asterisks indicate values significantly different from An-S at 0 h and between pepper varieties within same timepoint (* *p* < 0.05 and ** *p* < 0.01). All experiments were repeated at least three times and they generated similar results.

Next, we characterized the identified protein CbCN. Most NLRs are assumed to be cytoplasmic proteins as they contain no signal peptide or transmembrane region [32]. Consistently, CbCN lacked a signal peptide or transmembrane region. In order to examine the subcellular localization of CbCN in plants, we expressed N-terminal and C-terminal YFP-conjugated CbCN in chili pepper protoplasts. CbCN signals were predominantly observed in the cytosol (Figure 2A). In order to determine whether CbCN was involved in the disease response against anthracnose, we monitored the expression of *CbCN* by RT-qPCR after *C. acutatum* inoculation. The transcription level of *CbCN* was remarkably increased in both the susceptible variety An-S and the resistant variety PBC80 after *C. acutatum* inoculation (Figure 2B). Moreover, the expression of *CbCN* was dramatically higher in PBC80 than that in An-S (Figure 2B).

### 2.3. Enhanced Disease Resistance to C. acutatum in CbCN-Overexpressing Tobacco Plants

**Figure 3:**
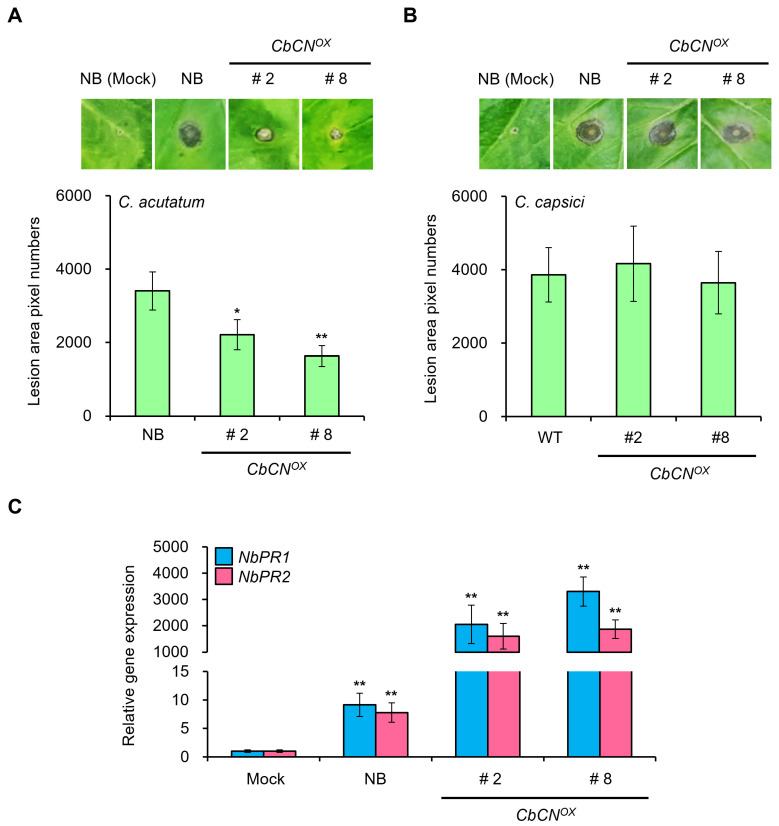
Anthracnose resistance of *CbCN*-expressing tobacco (*Nicotiana* *benthamiana*) plants to *C. acutatum*. (**A**,**B**) Anthracnose disease resistance assays of *CbCN*-overexpressing transgenic plants. Four-week-old *CbCN^OX^* and wild-type plants were inoculated with *C. acutatum* (**A**) and *C. capsici* (**B**) for the disease resistance assays, respectively. Images were captured at 6 days after inoculation and quantitative measurements of the disease area were obtained using the image-based plant disease phenotyping method. Values are expressed as means ± SDs. Asterisks indicate values statistically different from those of controls (* *p* < 0.05). (**C**) Analysis of the expression of *NbPR1* and *NbPR2* in *CbCN^OX^* plants using RT-qPCR. NbActin served as a control. Values are expressed as means ± SDs. Asterisks indicate values statistically different from those of controls (** *p* < 0.01). All experiments were repeated at least three times and they generated similar results.

In order to investigate the biological function of CbCN in plants, we generated transgenic tobacco plants (*Nicotiana benthamiana*) constitutively expressing *CbCN* (*CbCN^OX^*). The presence of the *CbCN* transgene was verified using RT-PCR (Supplementary Figure S1). In order to assess the involvement of CbCN in innate immunity against anthracnose, we analyzed the *CbCN^OX^* phenotype after *C. acutatum* inoculation. The *CbCN^OX^* leaves were more resistant to *C. acutatum* than the leaves of wild-type plants (Figure 3A). For accurate quantitative analysis, we measured the lesion area using the ImageJ software and confirmed that the susceptibility to *C. acutatum* was reduced in *CbCN^OX^* (Figure 3A). In order to examine whether CbCN specifically responds to *C. acutatum*, we inoculated *CbCN^OX^* plants with other *Colletotrichum* species such as *Colletotrichum capsici*. Unlike *C. acutatum* inoculation, *CbCN^OX^* plants showed the similar phenotype with wild-type plants after *C. capsici* inoculation (Figure 3B).

Next, we monitored the expression levels of genes related to innate immunity in *CbCN^OX^* and wild-type plants. The RT-qPCR assay indicated that the transcription levels of pathogenesis-related (*PR*) genes *NbPR1* and *NbPR2* were remarkably increased in *CbCN^OX^* (Figure 3C).

### 2.4. Attenuation of the Resistance to C. acutatum in Chili Pepper by CbCN Gene Silencing

Since *PR1* and *PR2* genes were upregulated by CbCN in tobacco, we examined whether these genes were associated with resistance to anthracnose in chili pepper varieties. The expression levels of *PR1* and *PR2* were significantly increased in An-S and PBC80 12 h after *C. acutatum* inoculation (Supplementary Figure S2). *PR1* expression was consistently higher in PBC80 than it was in An-S from 12 h to 48 h after disease induction (Supplementary Figure S2A). However, *PR2* expression level was consistently higher in PBC80 than it was in An-S from 24 h to 48 h after *C. acutatum* inoculation (Supplementary Figure S2B). These results suggest that a differential expression of *PR1* and *PR2* was involved in the innate immunity against anthracnose in chili pepper varieties.

**Figure 4:**
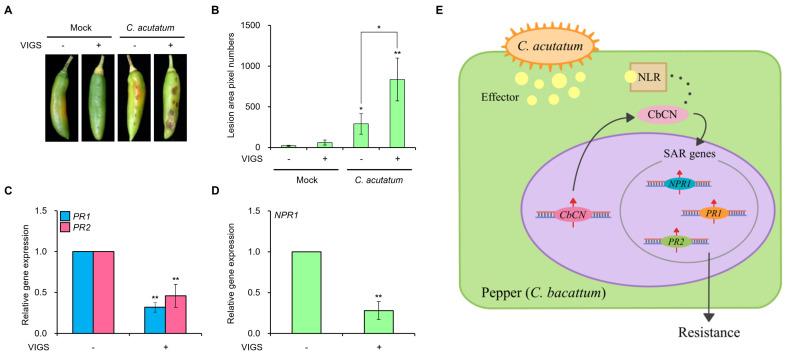
Anthracnose susceptibility and gene expression analysis in *CbCN*-silenced chili pepper fruits after *C. acutatum* inoculation. (**A**,**B**) Pathogen susceptibility assays of VIGS-mediated *CbCN*-silenced chili pepper fruits after *C. acutatum* inoculation. Chili pepper fruits of *CbCN*-silenced and wild-type plants were inoculated with *C. acutatum* or mock treatments. Images were captured after 6 days (**A**) and quantitative measurements of the disease area were obtained using the image-based plant disease phenotyping method (**B**). Values are expressed as means ± SDs. Asterisks indicate values statistically different from mock without VIGS and between present or absent of VIGS within *C. acutatum* inoculation (* *p* < 0.05 and ** *p* < 0.01). (**C**,**D**) Expression analysis of defense-response genes. Transcriptional levels of the SAR marker genes *PR1* and *PR2* (**C**) and SAR master regulator *NPR1* (**D**) were measured in *CbCN*-silenced chili pepper fruits after 6 days of *C. acutatum* inoculation using RT-qPCR. Actin served as a control. Values are expressed as means ± SDs. Asterisks indicate values statistically different from those of controls (** *p* < 0.01). (**E**) A working model of CbCN involvement in innate immunity against *C. acutatum*. The solid arrows indicate the role of CbCN verified in this study. The dashed line indicates hypothetical a process. All experiments were repeated at least three times and they generated similar results.

We investigated CbCN-mediated anthracnose resistance in chili pepper using the VIGS system to knock down the *CbCN* gene. The transcription of *CbCN* was reduced successfully as illustrated by RT-qPCR (Supplementary Figure S3A). We assessed the phenotype of *CbCN*-silenced plants inoculated with *C. acutatum* for 6 days. The susceptibility to *C. acutatum* increased significantly in *CbCN*-silenced chili pepper plants (Figure 4A). An image-based quantitative analysis indicated that the susceptibility to *C. acutatum* was more than 2.5-fold greater in the chili pepper fruits knocked down for *CbCN* than it was in the wild-type fruits (Figure 4B). However, *CbCN*-silencing did not affect the disease resistance to *C. capsici* in pepper fruit (Supplementary Figure S3B).

In order to determine whether CbCN upregulated *PR* genes in chili pepper, we performed RT-qPCR in *CbCN*-silenced chili pepper fruits inoculated with *C. acutatum*. The expressions of *PR1* and *PR2* were significantly decreased in *CbCN*-silenced chili peppers compared to that in wild-type fruits (Figure 4C). Moreover, nonexpressor of pathogenesis-related genes 1 (*NPR1*) transcription was downregulated significantly in *CbCN*-silenced chili pepper compared to that in wild-type plants (Figure 4D).

Taken together, these results indicated that *CbCN* is required to upregulate defense genes such as *PR1, PR2,* and *NPR1* in chili pepper.

## 3. Discussion

Chili pepper is a crop of crucial economic significance worldwide and anthracnose is one of the most important chili pepper disease. Previous genomic studies suggested that the NLR family is a central regulator of anthracnose resistance in chili pepper [1,2]. However, the *NLR* genes and their mechanisms of action in anthracnose resistance are largely unknown in chili peppers. Genomics approach were efficient to screen valuable genes in chili pepper. Therefore, we used genome-wide comparative analysis and identified *CbCN* in chromosome 12 of the resistant *C. baccatum* variety PBC80 as one of the candidate genes involved in anthracnose resistance to *C. acutatum* (Figure 1A).

Notably, unlike canonical NLRs, CbCN protein contained only the CC and NBS domains (Figure 1B,C). Transcriptome analyses have indicated that *NLR* genes were upregulated upon pathogen infection [33,34]. Moreover, *NLR* genes upregulated by specific pathogens were commonly associated with the innate immunity induced by those pathogens [35,36]. Therefore, the significant upregulation of *CbCN* mediated by *C. acutatum* in PBC80 suggested a role for *CbCN* in anthracnose resistance (Figure 2C). The NLR-mediated disease resistance is typically associated with a hypersensitive response and basal defenses. Similarly to typical NLRs, NLRs lacking the LRR domain were demonstrated to play a role in the recognition of effectors and the amplification of immune response [15]. Therefore, we generated *CbCN*-expressing tobacco (*Nicotiana benthamiana*) plants and analyzed the influence of *CbCN* on the innate immunity against anthracnose induced by *C. acutatum*. *CbCN^OX^* exhibited enhanced disease resistance to *C. acutatum* compared to that of wild-type plants (Figure 3A,B). The SAR-associated genes *PR1* and *PR2* were significantly induced in *CbCN^OX^* (Figure 3C). These results were confirmed in chili pepper using VIGS-mediated knockdown plants. In contrast to *CbCN^OX^* plants, *CbCN*-silenced chili peppers had an increased susceptibility toward *C. acutatum* (Figure 4A,B) and the expression levels of SAR genes such as *PR1*, *PR2*, and *NPR1* decreased compared to those present in wild-type chili peppers (Figure 4C,D). These results suggest a potential mechanism of action of CbCN, which acts by regulating the expression of genes involved in the SAR pathway (Figure 4E).

Thanks to the development of next-generation sequencing technologies, the NLR repertoires can be easily confirmed from various species [12]. Therefore, genomic analysis became the chosen method for identifying and characterizing NLR diversity. However, despite the tremendous amount of genomic information, the identification of atypical NLRs and their cellular function in disease resistance remains largely unexplored. It was proposed that truncated NLRs cooperated with typical NLRs for the effector recognition or downstream signaling. However, interaction pairs of typical and atypical NLRs have been poorly characterized. In this study, we identified a novel truncated NLR specific for *C. baccatum* and demonstrated that it enhanced the disease resistance by regulating the expression of genes related to SAR. Thus, our findings provide valuable knowledge required for one to understand how atypical NLRs transduce signals from *Colletotrichum* species to downstream effectors. This will provide insight into chili pepper plant breeding.

## 4. Materials and Methods

### 4.1. Plant Material and Growth Condition

In this study, the pepper varieties An-S and PBC80 and tobacco *Nicotiana benthamiana* (*N. benthamiana*) were used. They were sterilized in 70% ethanol for 1 min, followed by 5% sodium hypochlorite for 3 min, and then washed with sterilized distilled water. For seed germination, the seeds were sown on a half-strength Murashige and Skoog (MS) medium and incubated in a chamber with a 16 h light/8 h dark photoperiod at 28 °C. Depending on the experiment, the aseptic seedlings were grown continually in the chamber or transplanted into soil pots in a greenhouse with a 16 h light/8 h dark photoperiod at 28 °C.

### 4.2. Cloning of CbCN and the Conserved Domain Analysis

The full-length cDNA of *CbCN* (PHT28388) was cloned with the primers listed in Supplementary Table S1 from PBC80 through polymerase chain reaction (PCR). The cloned *CbCN* was inserted into the pDONR221 entry vector using Gateway BP Clonase II enzyme (Invitrogen, USA) according to the manufacturer’s instructions. The InterPro software tool was used to predict the conserved domains. Available online: https://www.ebi.ac.uk/interpro (accessed on 4 May 2020).

### 4.3. Subcellular Localization in Chili Pepper Protoplasts

For subcellular localization, the cDNA of *CbCN* was introduced into the pEarleyGate101 and 104 vectors using Gateway LR Clonase II enzyme (Invitrogen, USA) according to the manufacturer’s instructions. Protoplast isolation and polyethylene glycol (PEG) transfection were performed as described previously [37], with some modification. Briefly, the chili peppers were grown on a half-strength MS medium for 3–4 weeks. Primary leaves were cut into small pieces and incubated overnight in an enzyme solution containing 1.2% Cellulase RS (MBcell, Korea) and 0.3% Macerozyme R-10 (MBcell, Korea) in the dark for 12 h at 28 °C. The digested tissues were filtered gently through iron sieves and processed using sucrose density gradient centrifugation with 22% (*w*/*v*) sucrose to form pellets free of cell debris. A total of 40 µg of the desired DNA construct were mixed with 200 μL of protoplasts (4–6 × 10^4^) and PEG-mediated transfection was performed. The protoplasts were incubated in a six-well plate for 12 h at 28 °C with light. Fluorescence signals were detected using the Leica TCS SP8 confocal laser scanning microscope. All experiments were performed in a triplicate. Consistent results were obtained, and representative data were presented.

### 4.4. Gene Expression Analysis

Reverse transcription PCR (RT-PCR) and quantitative real-time PCR (RT-qPCR) were performed using SYBR Green Master Mix (Enzynomics, Korea) according to the manufacturer’s instructions. Briefly, cDNA synthesis was carried out using M-MLV reverse transcriptase (Promega, USA) on RNA isolated from chili pepper and tobacco plants. The cDNA was diluted 20-fold and PCR was performed using specific primers (Supplementary Table S1). The RT-qPCR was conducted on the MyiQ Real-Time PCR System (Bio-Rad, USA) under the following conditions: 40 cycles of denaturation at 95 °C for 10 s, annealing at 58 °C for 15 s, and extension at 72 °C for 30 s. Gene expression was quantified using the comparative Ct method. *Actin* was used as a calibration control to determine the expression of genes. All experiments were carried out independently at least three times.

### 4.5. Generation of Transgenic Tobacco Plants

In order to generate tobacco transgenic lines, *CbCN* transferred to a pEarleyGate201 binary vector using Gateway LR Clonase II enzyme (Invitrogen, USA) according to the manufacturer’s instructions. The recombinant construct was introduced into *Agrobacterium tumefaciens* (*A. tumefaciens*) strain LBA4404 by electroporation. The positive colonies were selected and cultured in YEP media supplemented with antibiotics. *Agrobacterium*-mediated leaf disk transformation was performed to generate transgenic tobacco plants as previously described [38]. The transformed plants were selected on MS plates supplemented with hygromycin and continuously cultivated to obtain homozygous T3 lines. The transgenic plants were verified by RT-PCR and two T3 lines were chosen for detailed analyses.

### 4.6. Virus-Induced Gene Silencing in Chili Pepper Plants

The virus-induced gene silencing (VIGS) target sequence was amplified using specific primers (Supplementary Table S1) and inserted into the pTRV2 VIGS vector. The pTRV1 and pTRV2/*CbCN* were transformed into an *A. tumefaciens* GV3101 strain using a freeze–thaw method. The cells containing the constructs were selected and used. VIGS was performed as previously described [39] with slight modifications. Briefly, suspensions of cells containing pTRV1 and pTRV2/*CbCN* were mixed at a 1:1 (*v*/*v*) ratio, precipitated by centrifugation for 10 min at 3000 × *g*, and resuspended in the same volume of the *Agrobacterium* infiltration buffer. The cells were infiltrated into chili pepper fruits and the inoculated plants were grown in the dark at 18 °C in 60% relative humidity for 48 h. The plants were then moved to the growth chamber with a 16 h light/8 h dark regime at 28 °C and incubated for 6 days. All experiments were performed in a triplicate. Consistent results were obtained, and representative data were presented.

### 4.7. Anthracnose Disease Resistance Assay

*C. acutatum* (KACC 40042) and *C. capsici* were cultured on petri dishes containing a potato agar medium for 10 days at 28 °C. Fungal suspensions were collected using a scalpel and passed through a cheesecloth. Their density was adjusted to 1 × 10^6^ conidia/mL using a hemocytometer. Anthracnose disease resistance was assayed using the pinning method with a toothpick for tobacco leaves and by microinjection for the chili pepper fruits, as previously described [40]. The inoculated plant tissues were incubated at 28 °C for 6 days. Quantitative measurements of the disease area were performed by image-based plant disease phenotyping as previously described [41]. All experiments were performed in a triplicate. Consistent results were obtained and representative data are presented.

### 4.8. Statistical Analysis

All experiments were repeated at least three times and the data were analyzed by *t*-test using GraphPad Prism 8.0 software. The asterisks indicate values statistically different (* *p* < 0.05 and ** *p* < 0.01).

## Data Availability

The data presented in this study are available in the article or the Supplementary Materials.

## Supplementary Materials

The following are available online at https://www.mdpi.com/article/10.3390/ijms22147672/s1, Figure S1: Verification of *CbCN*-overexpression transgenic plant lines, Figure S2: Expression analysis of *PR1* and *PR2* after *C. acutatum* inoculation, Figure S3. Analysis of the *CbCN*-silencing efficiency and disease resistance to *C. capsici* in *CbCN* knockdown chili pepper fruits, Table S1. List of primers used in this study.

## References

[1] Kim S., Park M., Yeom S.I., Kim Y.M., Lee J.M., Lee H.A., Seo E., Choi J., Cheong K., Kim K.T. (2014). Genome sequence of the hot pepper provides insights into the evolution of pungency in Capsicum species. Nat. Genet..

[2] Kim S., Park J., Yeom S.I., Kim Y.M., Seo E., Kim K.T., Kim M.S., Lee J.M., Cheong K., Shin H.S. (2017). New reference genome sequences of hot pepper reveal the massive evolution of plant disease-resistance genes by retroduplication. Genome Biol..

[3] Dangl J.L., Horvath D.M., Staskawicz B.J. (2013). Pivoting the plant immune system from dissection to deployment. Science.

[4] von Moltke J., Ayres J.S., Kofoed E.M., Chavarria-Smith J., Vance R.E. (2013). Recognition of bacteria by inflammasomes. Annu. Rev. Immunol..

[5] Jones J.D., Vance R.E., Dangl J.L. (2016). Intracellular innate immune surveillance devices in plants and animals. Science.

[6] Dodds P.N., Rathjen J.P. (2010). Plant immunity: Towards an integrated view of plant-pathogen interactions. Nat. Rev. Genet..

[7] Spoel S.H., Dong X. (2012). How do plants achieve immunity? Defence without specialized immune cells. Nat. Rev. Immunol..

[8] Wang J., Wang J., Hu M., Wu S., Qi J., Wang G., Han Z., Qi Y., Gao N., Wang H.W. (2019). Ligand-triggered allosteric ADP release primes a plant NLR complex. Science.

[9] Hammond-Kosack K.E., Jones J.D. (1996). Resistance gene-dependent plant defense responses. Plant Cell.

[10] Durrant W.E., Dong X. (2004). Systemic acquired resistance. Annu. Rev. Phytopathol..

[11] Borrelli G.M., Mazzucotelli E., Marone D., Crosatti C., Michelotti V., Vale G., Mastrangelo A.M. (2018). Regulation and Evolution of NLR Genes: A Close Interconnection for Plant Immunity. Int. J. Mol. Sci..

[12] Monteiro F., Nishimura M.T. (2018). Structural, Functional, and Genomic Diversity of Plant NLR Proteins: An Evolved Resource for Rational Engineering of Plant Immunity. Annu. Rev. Phytopathol..

[13] Lolle S., Stevens D., Coaker G. (2020). Plant NLR-triggered immunity: From receptor activation to downstream signaling. Curr. Opin. Immunol..

[14] Meyers B.C., Kozik A., Griego A., Kuang H., Michelmore R.W. (2003). Genome-wide analysis of NBS-LRR-encoding genes in Arabidopsis. Plant Cell..

[15] Baggs E., Dagdas G., Krasileva K.V. (2017). NLR diversity, helpers and integrated domains: Making sense of the NLR IDentity. Curr. Opin. Plant Biol..

[16] Staal J., Kaliff M., Dewaele E., Persson M., Dixelius C. (2008). RLM3, a TIR domain encoding gene involved in broad-range immunity of Arabidopsis to necrotrophic fungal pathogens. Plant J..

[17] Wang Y., Zhang Y., Wang Z., Zhang X., Yang S. (2013). A missense mutation in CHS1, a TIR-NB protein, induces chilling sensitivity in Arabidopsis. Plant J..

[18] Zhao T., Rui L., Li J., Nishimura M.T., Vogel J.P., Liu N., Liu S., Zhao Y., Dangl J.L., Tang D. (2015). A truncated NLR protein, TIR-NBS2, is required for activated defense responses in the exo70B1 mutant. PLoS Genet..

[19] Roth C., Ludke D., Klenke M., Quathamer A., Valerius O., Braus G.H., Wiermer M. (2017). The truncated NLR protein TIR-NBS13 is a MOS6/IMPORTIN-alpha3 interaction partner required for plant immunity. Plant J..

[20] Liang W., van Wersch S., Tong M., Li X. (2019). TIR-NB-LRR immune receptor SOC3 pairs with truncated TIR-NB protein CHS1 or TN2 to monitor the homeostasis of E3 ligase SAUL1. New Phytol..

[21] Liang W., Tong M., Li X. (2020). SUSA2 is an F-box protein required for autoimmunity mediated by paired NLRs SOC3-CHS1 and SOC3-TN2. Nat. Commun..

[22] Barragan A.C., Collenberg M., Wang J., Lee R.R.Q., Cher W.Y., Rabanal F.A., Ashkenazy H., Weigel D., Chae E. (2021). A Truncated Singleton NLR Causes Hybrid Necrosis in Arabidopsis thaliana. Mol. Biol. Evol..

[23] Nandety R.S., Caplan J.L., Cavanaugh K., Perroud B., Wroblewski T., Michelmore R.W., Meyers B.C. (2013). The role of TIR-NBS and TIR-X proteins in plant basal defense responses. Plant Physiol..

[24] Than P.P., Prihastuti H., Phoulivong S., Taylor P.W., Hyde K.D. (2008). Chilli anthracnose disease caused by Colletotrichum species. J. Zhejiang Univ. Sci. B.

[25] Kim H.G., Bae J.H., Jastrzebski Z., Cherkas A., Heo B.G., Gorinstein S., Ku Y.G. (2016). Binding, Antioxidant and Anti-proliferative Properties of Bioactive Compounds of Sweet Paprika (*Capsicum annuum* L.). Plant Foods Hum. Nutr..

[26] Surh Y.J. (2002). More than spice: Capsaicin in hot chili peppers makes tumor cells commit suicide. J. Natl. Cancer Inst..

[27] Ridzuan R., Rafii M.Y., Ismail S.I., Mohammad Yusoff M., Miah G., Usman M. (2018). Breeding for Anthracnose Disease Resistance in Chili: Progress and Prospects. Int. J. Mol. Sci..

[28] Albrecht E., Zhang D., Mays A.D., Saftner R.A., Stommel J.R. (2012). Genetic diversity in Capsicum baccatum is significantly influenced by its ecogeographical distribution. BMC Genet..

[29] Manzur J.P., Fita A., Prohens J., Rodriguez-Burruezo A. (2015). Successful Wide Hybridization and Introgression Breeding in a Diverse Set of Common Peppers (*Capsicum annuum*) Using Different Cultivated Aji (*C. baccatum*) Accessions as Donor Parents. PLoS ONE.

[30] Schulze-Lefert P., Panstruga R. (2011). A molecular evolutionary concept connecting nonhost resistance, pathogen host range, and pathogen speciation. Trends Plant Sci..

[31] Lee J., Hong J.-H., Do J.W., Yoon J.B. (2010). Identification of QTLs for resistance to anthracnose to two *Colletotrichum* species in pepper. J. Crop. Sci. Biotechnol..

[32] McHale L., Tan X., Koehl P., Michelmore R.W. (2006). Plant NBS-LRR proteins: Adaptable guards. Genome Biol..

[33] Lai Y., Eulgem T. (2018). Transcript-level expression control of plant NLR genes. Mol. Plant Pathol..

[34] Chakraborty S., Britton M., Martinez-Garcia P.J., Dandekar A.M. (2016). Deep RNA-Seq profile reveals biodiversity, plant-microbe interactions and a large family of NBS-LRR resistance genes in walnut (*Juglans regia*) tissues. AMB Express.

[35] Eulgem T., Weigman V.J., Chang H.S., McDowell J.M., Holub E.B., Glazebrook J., Zhu T., Dangl J.L. (2004). Gene expression signatures from three genetically separable resistance gene signaling pathways for downy mildew resistance. Plant Physiol..

[36] Bartsch M., Gobbato E., Bednarek P., Debey S., Schultze J.L., Bautor J., Parker J.E. (2006). Salicylic acid-independent ENHANCED DISEASE SUSCEPTIBILITY1 signaling in Arabidopsis immunity and cell death is regulated by the monooxygenase FMO1 and the Nudix hydrolase NUDT7. Plant Cell.

[37] Yoo S.D., Cho Y.H., Sheen J. (2007). Arabidopsis mesophyll protoplasts: A versatile cell system for transient gene expression analysis. Nat. Protoc..

[38] Sohn S., Choi M.S., Kim K., Lomonossoff G. (2011). The epigenetic phenotypes in transgenic Nicotiana benthamiana for CaMV 35S-GFP are mediated by spontaneous transgene silencing. Plant Biotechnol. Rep..

[39] Tian S.L., Li L., Chai W.G., Shah S.N., Gong Z.H. (2014). Effects of silencing key genes in the capsanthin biosynthetic pathway on fruit color of detached pepper fruits. BMC Plant Biol..

[40] Yoon J.B., Park H.G. (2001). Screening method for resistance to pepper fruit anthracnose: Pathogen sporulation, inoculation methods related to inoculum concentrations and post-inoculation environment. J. Korea Soc. Hort. Sci..

[41] Mutka A.M., Fentress S.J., Sher J.W., Berry J.C., Pretz C., Nusinow D.A., Bart R. (2016). Quantitative, Image-Based Phenotyping Methods Provide Insight into Spatial and Temporal Dimensions of Plant Disease. Plant Physiol..

