# Supplementary material for: The Capsicum baccatum-Specific Truncated NLR Protein CbCN Enhances the Innate Immunity against Colletotrichum acutatum
Source: Int J Mol Sci. 2021 Jul 18;22(14):7672. doi: 10.3390/ijms22147672 (PMC8306327; doi:10.3390/ijms22147672)
Supplement: Supplementary file 1 [file ijms-22-07672-s001.zip › ijms-1281840-supplementary.pdf]

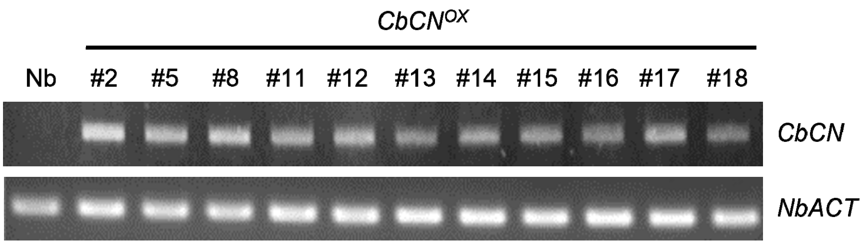

Figure S1. Verification of *CbCN*-overexpression transgenic plant lines. Expression of *CbCN* was analysis at *CbCN*^{OX} and wild-type tobacco plants by RT-PCR. NbActin served as a control.

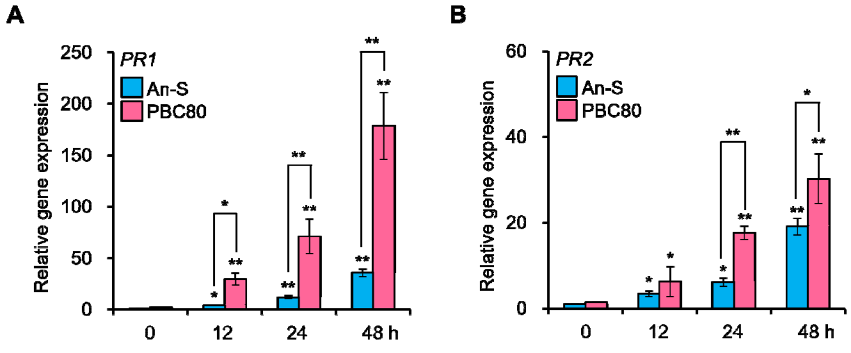

Figure S2. Expression analysis of *PR1* and *PR2* after *C. acutatum* inoculation. (A,B) Expression levels of *PR1* (A) and *PR2* (B) were measured after *C. acutatum* inoculation for the indicated times using RT-qPCR. Actin served as a control. Values represent means ± SDs. Asterisks indicate values statistically different from An-S at 0 hour and between pepper varieties within same timepoint (* $P < 0.05$ and ** $P < 0.01$). All experiments were repeated at least three times, and they generated similar results.

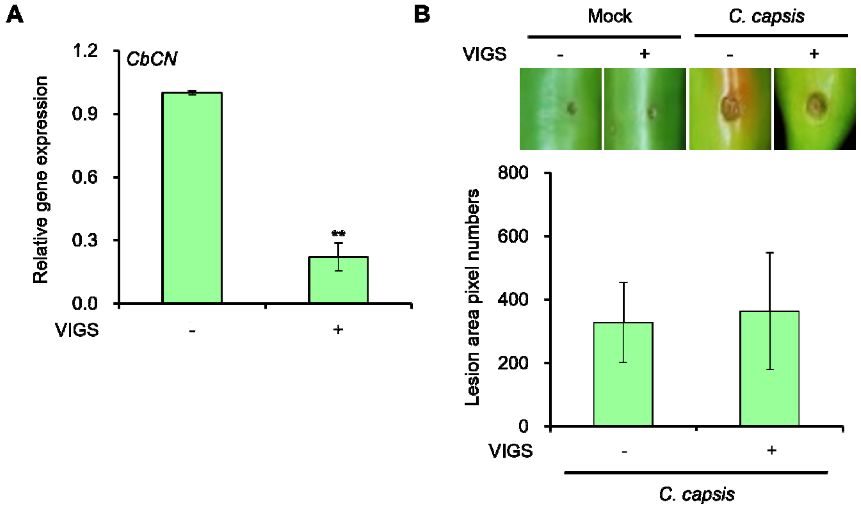

Figure S3. Analysis of the *CbCN*-silencing efficiency and disease resistance to *C. capsici* in *CbCN* knockdown pepper fruits. (A) Relative transcript levels of *CbCN* in *CbCN*-silenced pepper fruits and control. Relative *CbCN* transcript levels were determined by RT-qPCR. Actin served as a control. Values represent means ± SDs. Asterisks indicate values statistically different from controls (** P < 0.01). (B) Pathogen susceptibility assays of VIGS-mediated *CbCN*-silenced pepper fruits after *C. capsici* inoculation. Pepper fruits of *CbCN*-silenced and wild-type plants were inoculated with *C. capsici* or mock inoculations. Images were captured after 6 days, and quantitative measurements of the disease area were obtained using the image-based plant disease phenotyping method. Values are expressed as means ± SDs. All experiments were repeated at least three times, and they generated similar results.

Gene	Primer sequence	Purpose
CbCN	F: AAAAAGCAGGCTAAATGGCTCATGCAAGTGTGG	Cloning
	R: AGAAAGCTGGGTATCAACGGACGATGTACAA	Cloning
	R: AGAAAGCTGGGTAACGGACGATGTACAAGGG	Cloning
	F: TCTAGAATGGCTCATGCAAGTGTC	VIGS
	R: GGTACCAACTTCAGTTACGCTTAGTT	VIGS
	F: GCCTGGGCTACTATTTCGCA	qRT-PCR
	R: CATAGTCTCACGGCATCCCA	qRT-PCR
PR1	F: GAGGACAACGTCCGTATGGT	qRT-PCR
	R: AACTCCAGTTACTGCACCATTA	qRT-PCR
PR2	F: CTACTTAAGCTTTGCAAGCACCA	qRT-PCR
	R: AGATCTCTTTCCTCATCGTCACTT	qRT-PCR
NPR1	F: TGAACAGGATTCAATAGAAGTGGA	qRT-PCR
	R: AAATCCAGCTCAAGTACCTCATTC	qRT-PCR
ACT	F: GTGCTGAGAGATTCCGTTGC	qRT-PCR
	R: ATGGTTGAGCCACCACTGAG	qRT-PCR
NbPR1	F: GTGCCCAAAATTCTCAACAAGACT	qRT-PCR
	R: AAATCGCCACTTCCCTCAGC	qRT-PCR
NbPR2	F: CAACATAACCTTCCACTCTTAGCCA	qRT-PCR
	R: CATAGAATCCAAAAGGGCATCAAAAAGA	qRT-PCR
NbACT	F: CCCAGATGGGCAGGTGATCA	qRT-PCR
	R: GAGTTGTATGTGGTCTCGTGGATTC	qRT-PCR

Table S1. List of primers used in this study.
